# An Assessment of Quality-of-Life Following Tissue Expansion in Pediatric Patients

**DOI:** 10.1177/22925503231217516

**Published:** 2023-12-12

**Authors:** Dara Rykiss, Rebecca Courtemanche, Sally Hynes

**Affiliations:** 1 Division of Plastic Surgery, Department of Surgery, 8166 University of British Columbia (DR, SH), 37210 BC Children's Hospital Research Institute, Vancouver, BC, Canada (SH)

**Keywords:** quality of life, child, tissue expansion, skin, surveys, questionnaires, qualité de vie, enfant, expansion tissulaire, peau, enquêtes, questionnaires

## Abstract

**Introduction:** There is limited data on the effects of tissue expansion (TE) surgery on quality-of-life (QOL) in pediatric patients. Evidence-based information may help clinicians, patients, and their families with treatment decision-making and post-operative expectations. This study explores QOL following TE in pediatric patients. **Methods:** A post-intervention QOL survey and retrospective chart review was performed. Patients who underwent TE at a pediatric tertiary hospital between October 2004 and March 2020 completed the Glasgow Children's Benefit Inventory or the Glasgow Benefit Inventory. Total scores range from −100 (worsened QOL) to +100 (improved QOL). Participants were also asked if they would recommend TE to other patients with the same indication. **Results:** The response rate was 38% (n = 20; 14 females, 6 males). The median QOL score was +17[−2,+49]. Higher QOL scores were found for TE indications of scar (+49) and alopecia (+40), compared to giant congenital melanocytic nevus (−2). Higher scores were also associated with shorter time in active treatment (*r* = −0.65) and fewer complications (*r* = −0.56). 18/20 participants would recommend TE. Participants whose QOL improved (n = 11, 55%) reported increased self-confidence, fitting in with peers, and improved function. Participants with unchanged or decreased QOL (n = 9, 45%) described a negative experience with residual scarring. However, 7/9 with unchanged or decreased QOL would still recommend TE. **Conclusions:** This preliminary descriptive study demonstrated variable QOL following TE. Patient and treatment-related factors impact QOL outcomes. Despite the range in QOL outcomes, the majority of participants would recommend TE. Further research evaluating QOL following TE is necessary to better understand this relationship.

## Résumé

**Introduction :** On ne dispose que de données limitées sur les effets de l’expansion tissulaire (ET) sur la qualité de vie (QdV) après chirurgie chez des patients pédiatriques. Une information basée sur des données probantes peut aider les cliniciens, les patients et leurs familles dans la prise de décision thérapeutique et sur les attentes postopératoires. Cette étude explore la QdV après ET chez des patients pédiatriques. **Méthodes :** Une enquête sur la qualité de vie postopératoire et une revue rétrospective des dossiers ont été réalisées. Les patients qui avaient subi une ET dans un hôpital pédiatrique de niveau tertiaire entre octobre 2004 et mars 2020 ont rempli le GCBI (Glasgow Children's Benefit Inventory) ou le GBI (Glasgow Benefit Inventory). Les cotes totales étaient comprises entre −100 (QdV aggravée) et +100 (QdV améliorée). On a également demandé aux participants s’ils recommanderaient l’ET à d’autres patients ayant la même indication. **Résultats :** Le taux de réponse a été de 38% (n = 20, 14 répondants de sexe féminin et 6 de sexe masculin). La cote médiane de QdV était +17 (−2 à + 49). Les cotes de QdV les plus élevées ont été attribuées aux indications d’ET pour cicatrice (+49) et alopécie (+40) comparativement au nævus mélanocytaire congénital géant (−2). Les cotes les plus élevées ont été associées au temps de traitement actif le plus court (r = −0,65) et au minimum de complications (r = −0,56). Dix-huit participants sur 20 recommanderaient l’ET. Les participants dont la QdV a été améliorée (n = 11, 55%) ont décrit une amélioration de la confiance en soi, de l’assimilation parmi les pairs et d’un meilleur fonctionnement. Les participants à la QdV inchangée ou diminuée (n = 9, 45%) ont décrit une expérience négative avec les cicatrices résiduelles. Cependant, 7 patients sur 9 avec une QdV inchangée ou diminuée recommanderaient toujours l’ET. **Conclusions :** Cette étude descriptive préliminaire a mis en évidence une QdV variable après ET. Les facteurs liés aux patients et aux traitements influencent les résultats sur la QdV. Malgré la plage des résultats de la QdV, la majorité des participants recommanderaient l’ET. Des recherches supplémentaires évaluant la QdV après ET sont nécessaires pour mieux comprendre cette relation.

## Introduction

Since the introduction of tissue expansion (TE) to pediatric clinical practice, surgeons have been increasingly using this surgical technique. TE has allowed surgeons to achieve previously unattainable functional and aesthetic goals in the reconstruction of large and challenging skin lesions, including congenital melanocytic nevi (CMN), burn scars, and alopecia.^1^ TE can provide large flaps of highly vascularized local tissue, with the best potential match in color, texture, thickness, and hair-bearing characteristics.^2^

TE aims to improve a patient's functional and/or psychosocial well-being. However, despite its advantages, TE is a time-consuming and intensive procedure, which can be burdensome on a patient, their family, and the clinical team. The procedure requires a minimum of 2 operations, in addition to weekly expander injections. Altogether, a single session typically takes around 3 months.^3^ The process involves a temporary dramatic physical deformity that may be distressing and negatively impact patients’ psychosocial wellbeing.^4^ TE also has a high rate of complications including infection, tissue necrosis, dehiscence, expander extrusion, and scarring.^1,2,5,6^ Since TE is undertaken when there are few feasible alternatives, this high rate of complications may be accepted.^6^ With these challenges in mind, pre-operative counselling, expectation management, and a thorough informed consent process, as well as a high level of commitment from the patient and family, are considered to be vital components of the treatment process.^1,5^

The current literature on pediatric TE reports traditional outcome measures (eg, operative complications and overall reconstructive success) and focuses on treatment details (eg, expander shape and volume). There is a paucity of data on the effects of TE on QOL following surgery. When discussing the risks and benefits of TE with patients and families, one of the most challenging aspects for the clinical team is a lack of information on how it will affect the child's quality-of-life (QOL). Health-related QOL has been described as a multidimensional concept encompassing physical, psychological, and social aspects,^7^ and is often the way in which patients and their families perceive and evaluate the impact and success of an intervention.^8^ Evidence-based information may help clinicians, patients, and their families with treatment decision-making and establishing expectations for the post-operative period.^9^ This study explores QOL following TE in pediatric patients.

## Methods

This study was approved by the University's Research Ethics Board (#H20-01466). The study was designed as a post-intervention QOL survey and retrospective chart review. All patients who underwent TE at a pediatric tertiary hospital from October 1, 2004 to March 15, 2020 were invited to participate. Informed consent was obtained for the QOL survey and medical chart review.

### QOL Survey

The Glasgow Children's Benefit Inventory (GCBI) and Glasgow Benefit Inventory (GBI) are validated questionnaires used to assess post-intervention QOL (Supplemental File).^10,11^ To assess health-related QOL following TE, participants were invited to complete the GCBI or GBI as determined by their age at the time of questionnaire administration. For participants who were under 18 years of age, their parents or caregivers completed the GCBI on their behalf. The questions are classified into 4 domains for the GCBI (emotion, physical health, learning, vitality) and 3 domains for the GBI (general, physical, social health). Each question is rated on a 5-point Likert scale ranging from “much worse” to “no change” to “much better.” The GCBI values range from −2 to +2 and the GBI values range from +1 to +5, with the latter converted to a −2 to +2 range. Total scores were calculated by summing up all points, dividing by the number of questions (24 for GCBI, 18 for GBI), subtracting 3 (for GBI only), and multiplying by 50 to achieve a score that ranged from −100 through 0 to +100. Scores were also calculated for each domain. In addition to the questionnaire, all participants were asked whether they would recommend TE to another child with the same surgical indication and to explain why or why not.

### Chart Review

Data collected from the medical charts of participants included: patient demographics, TE characteristics, details of the expansion process, operative details, and complications. The term “tissue expansion session” was used to define one cycle of expander implantation, expansion, and removal, ending with a full or partial repair of a defect.

### Data Analysis

Patient characteristics, treatment details, and complications were described and tabulated for the study population. GCB/GCBI QOL scores were described using medians and interquartile ranges (IQRs). Trends in the QOL scores were assessed visually and via Spearman correlation tests. Reasons for or against participants recommending TE were analyzed qualitatively.

## Results

In the study period from October 2004 to March 2020, pediatric patients were treated and followed by one of the plastic surgeons at our hospital. Of the 53 patients who met the inclusion criteria and were invited to participate in the study, 20 consented to participate and were included in the final sample. Of those that did not participate, 17 never consented after a few attempts to discuss the study, 14 could not be reached by post mail or telephone, and 2 declined. Of the 2 patients who declined, 1 felt their TE experience was in the context of a much larger procedure and their experience would not add value to the study, and the second did not provide a reason. Therefore, the response rate was 38% (n = 20). Fourteen participants were female, and 6 were males. One participant did not consent to a chart review, and was excluded from further analyses.

Patient characteristics are shown in Table 1. At the time of GBI/GCBI completion, 11 participants were children and 9 were adults. The most frequent surgical indications were giant congenital melanocytic nevi (GCMN) (n = 8) and scar (n = 6) and the most frequent lesion locations were on the extremities (n = 8) and scalp (n = 7). Lesion sizes varied from “2.5 × 1 cm” to “40%-50% total body surface area.” Our sample included patients who required TE as a setup for another procedure (eg, TE prior to closure of a sternal cleft). Some patients underwent further reconstructive surgeries following TE such as serial excision and scar revision.

**Table 1.** Patient Characteristics and Individual GCBI/GBI Scores.

Patient	Sex	Diagnosis	Location	Relevant medical history	Time from last TE to GBI/GCBI completion (year)	Age at GBI/GCBI completion (year)	Total GCBI/GBI score	Recommends TE (Y/N)
1	F	Scar	Scalp	GCMN, hypertrophic scar post TE	7.8	25	53	Y
2	M	Alopecia	Scalp	Kerion and dermatophytic granuloma, scalp wound secondary to abscess I & D	10	23	61	Y
3	M	GCMN	Face	N	3.3	8	0	Y
4	F	GCMN	UE	N	nd	11	−19	Y
5	F	Scar	Trunk, UE, LE	Hot oil burn	7.2	11	56	Y
6	F	Scar	LE	Iatrogenic chemical burn	9.7	11	65	Y
7	F	CD	Trunk (Breast)	Poland Syndrome, hypoplastic right chest wall	7.3	19	61	Y
8	F	CD	Scalp	Cutis aplasia congenita	4.1	18	−17	Y
9	M	Scar	LE	Hypertrophic scar secondary to necrotizing fasciitis	10.9	27	14	Y
10	F	GCMN	LE	N	4.8	10	17	Y
11	F	CD	Trunk	PHACES Syndrome, Sternal cleft	6	17	20	Y
12	M	Scar	Scalp	Bilateral sensorineural hearing loss; atrophic scarring post cochlear implant infection	9.4	20	8	Y
13	M	nd	nd	nd	nd	24	−33	N
14	M	GCMN	Scalp	N	15.4	20	36	Y
15	F	GCMN	UE	N	0.4	5	−13	Y
16	F	GCMN	LE	N	4.8	9	−4	Y
17	F	GCMN	Scalp	N	8.7	9	0	Y
18	M	Alopecia	Scalp	Caput succedaneum, ischemic injury and necrosis of hair follicles	14.7	19	18	N
19	F	GCMN	Trunk, LE	N	8.8	12	−31	Y
20	F	Scar	Trunk	Scarring secondary to full thickness burns	15.5	30	44	Y

Abbreviations: M, male; F, female; GCMN, giant congenital melanocytic nevus; CD, congenital disorder; UE, upper extremity; LE, lower extremity; BSA, body surface area; nd, no data; N, none.

### Treatment Details

Tissue expander treatment details are shown in Table 2. Participants underwent an average of 2.2 sessions (range, 1-5) with an average of 3.4 expanders for the entire treatment (range, 1-16). The median age at first expander insertion was 5 years (range, 1-17) and the average total time spent in active TE treatment sessions was 3.6 months (range, 1-17).

**Table 2.** Tissue Expansion Treatment Details.

Patient	Total sessions (count)	Location of expansions	Age at first expander insertion (years)	Age at last expander removal (years)	Duration of entire TE tx (months)	Time spent in active TE sessions (months)	Total expanders over entire tx (count)	Expanders per session (average)
1	1	Home	17	17	4	4	1	1
2	1	CP	12	13	4	4	2	2
3	4	BCCH	1	5	53	16	4	1
4	4	BCCH, Home	1	nd	nd	nd	5	1
5	4	BCCH, Home	2	5	26	nd	9	3
6	1	Home	1	2	2	4	2	2
7	1	nd	12	12	1	1	1	1
8	3	BCCH, Home	10	15	58	17	7	2
9	1	BCCH	16	17	4	4	2	2
10	1	BCCH, Home	5	5	82	4	1	1
11	1	BCCH, Home	11	11	4	4	2	2
12	1	BCCH	10	11	5	6	1	1
13	nd	nd	nd	nd	nd	nd	nd	nd
14	3	nd	2	6	36	7	5	2
15	2	BCCH, Home	3	nd	16	11	3	2
16	4	CP	1	5	46	13	4	1
17	1	BCCH	1	1	1	1	1	1
18	1	nd	5	5	4	4	2	2
19	5	BCCH, Home	1	4	40	17	16	3
20	2	BCCH	14	15	7	7	3	2

Abbreviations: BCCH, BC Children's Hospital; CP, Community Physician; nd, no data; tx, treatment.

### Complications

Thirteen patients (68%) had one or more complications. The average number of complications per patient was 3 (range, 0-11). There were similar rates of expander and flap complications (80% and 85%, respectively). Expander complications included exposure (20%), delayed wound healing (20%), infection (15%), port failure/displacement (15%), and deflation (10%). Flap complications included dehiscence (25%), necrosis (30%), infection (20%), delayed wound healing (5%), and temporary ischemia (5%). Complications were most frequently managed with observation or early expander removal.

### Health-Related QOL GCB/GCBI Scores and Trends

**Figure 1.**
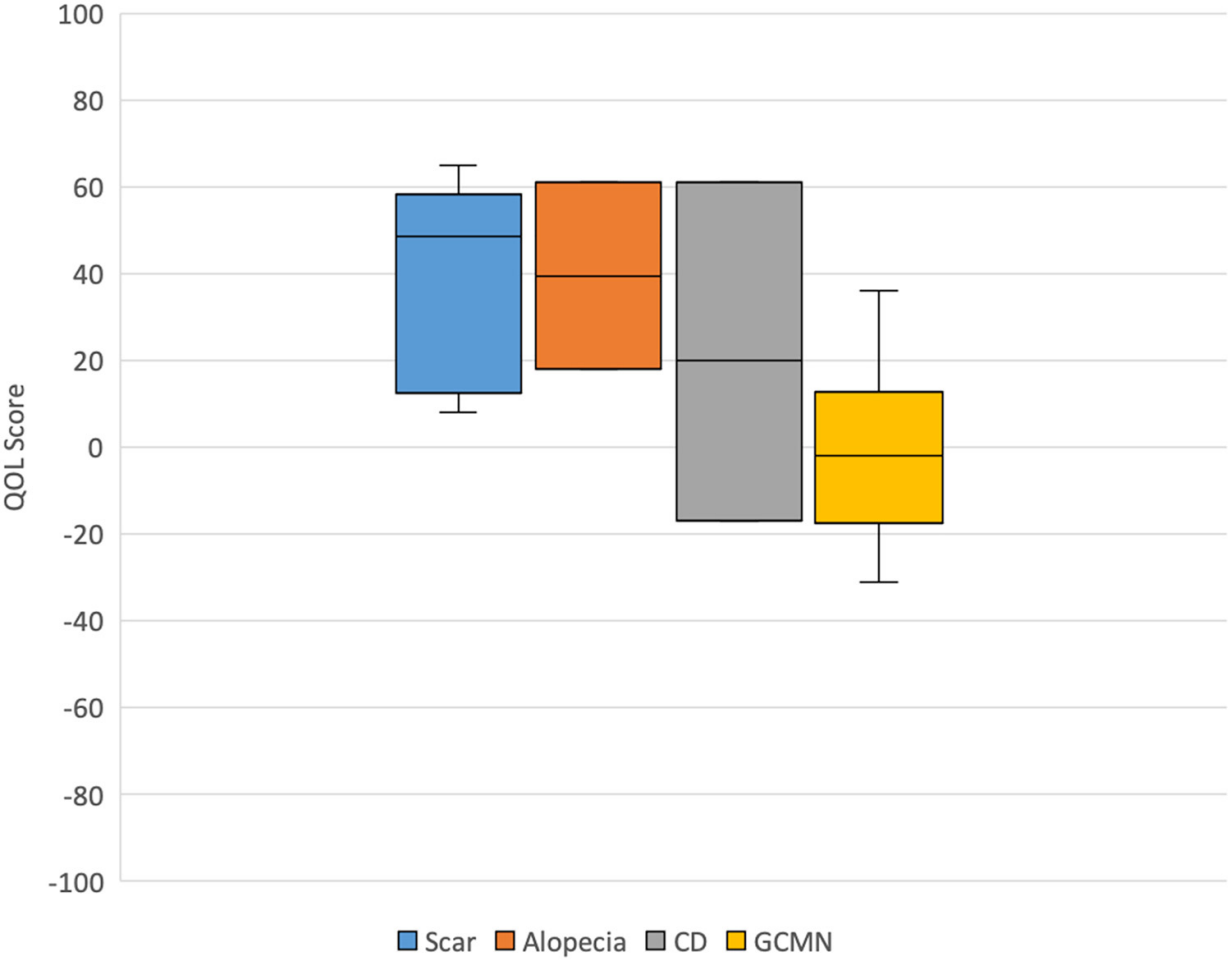
Quality-of-life score by indication for tissue expansion.

The median QOL score was +17 [IQR −2,  + 49]. The median QOL score in pediatric participants was 0 [IQR −15,  + 18], and the median QOL score in adult participants was +36 [IQR +14,  + 53]. The median scores for the emotion, physical health, and vitality domains of the GCBI were 0 [IQR −15,  + 13; −13,  + 22; −5,  + 18]. The median scores for the learning, general, social, and physical domains of the GBI were +4 [IQR −8,  + 27],  + 29 [IQR 17,  + 54], 0 [IQR 0,  + 33], and +33 [IQR +17,  + 67] respectively (Figure 1).

The highest median QOL scores were found for patients with TE indications for scar (49) and alopecia (40), while the lowest median QOL score was for patients with GCMN (−2). No significant correlation was found between QOL and anatomical location of the lesion. Size of lesion for the GCMN subgroup was not consistently reported with objective terminology, and thus a subgroup analysis could not be performed.

**Figure 2.**
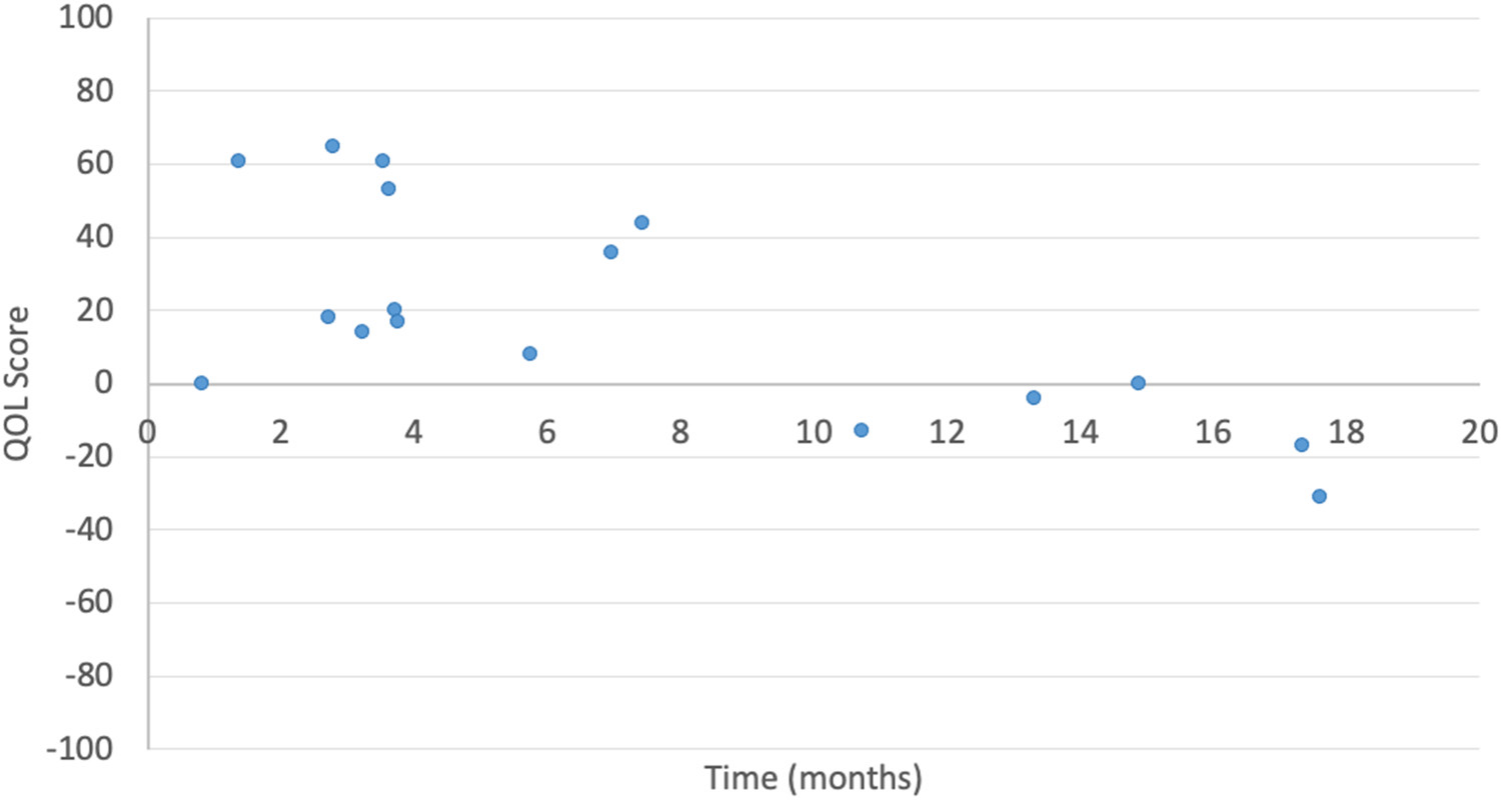
Quality-of-life score by total time in active treatment.

Higher QOL scores following TE tended to be associated with shorter time in active treatment (*r* = −0.65, *P* < .005) (Figure 2), fewer total TE sessions (*r* = −0.54, *P* = .018), and an older age at first expander insertion (*r* = 0.50, *P* = .029). No significant correlations were found between QOL and anatomical location of lesion, number of expanders, or time between TE and questionnaire administration.

**Figure 3.**
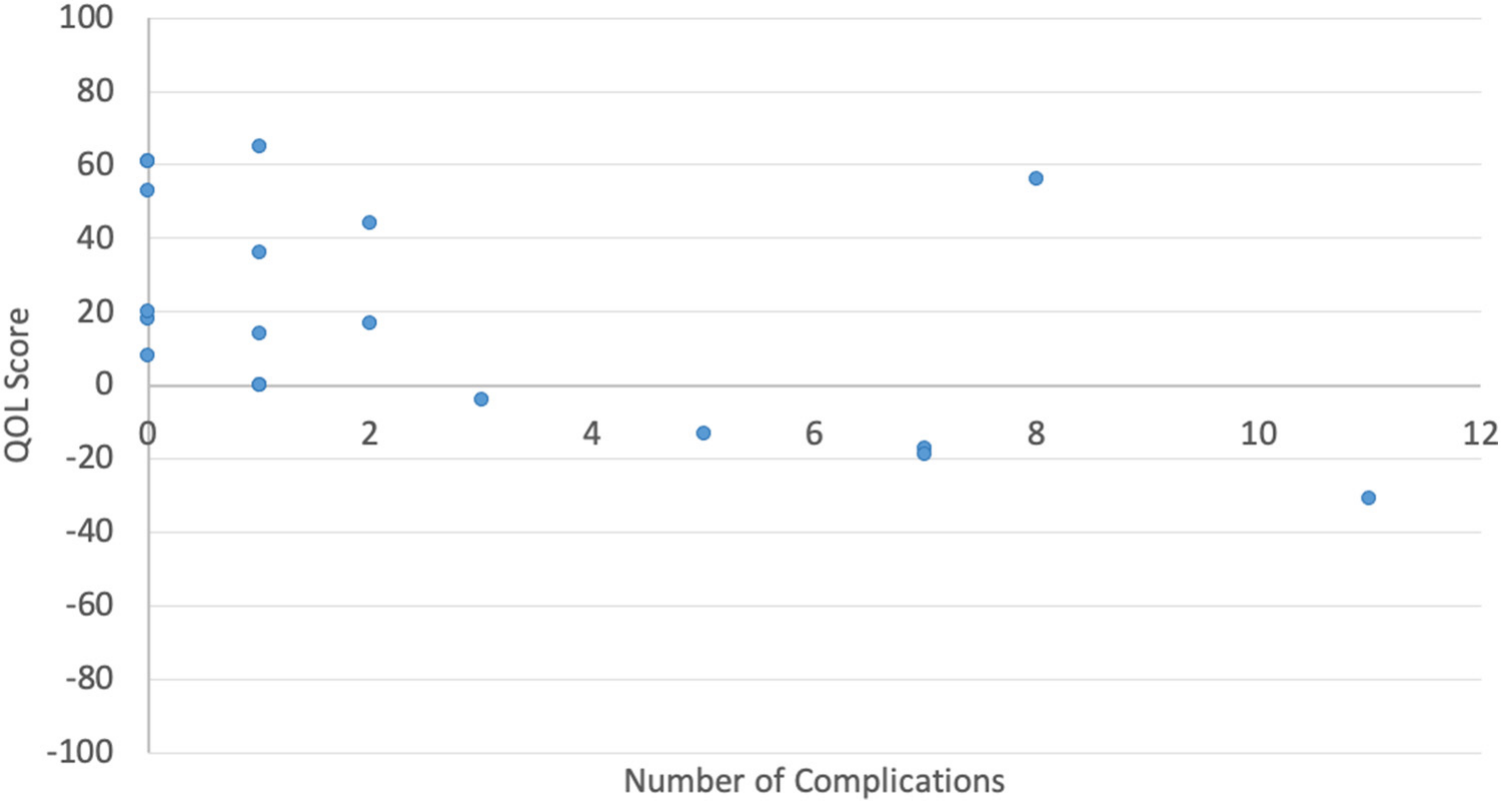
Quality-of-life score by total number of complications.

Participants without complications had QOL scores ranging from +8 to +61 while those with complications had QOL scores ranging from −33 to +65. Participants who had a higher number of complications tended to have a lower QOL score (*r* = −0.56, *P* = .013) (Figure 3), and this trend was more evident for patients with GCMN (*r* = −0.88, *P* = .004).

### Participant Recommendations

Eighteen out of 20 participants would recommend TE to another patient with a similar surgical indication. Participants whose QOL improved (n = 11, 55%) reported increased self-confidence, fitting in with peers, and improved functional outcomes. Participants whose QOL did not change or decreased (n = 9, 45%) described a negative experience with residual scarring. However, 7 out of 9 with unchanged or decreased QOL would still recommend TE.

Patients reported feelings towards their scars varied. One participant's caregiver stated that her daughter “likes wearing clothes that might show her scar as she's not ashamed of it.” Another noted that “people ask if she's been burned due to the severity of her stretched scars” and that “she's struggling with this recently emotionally.” For the two participants who did not recommend TE: one had residual scars on their head following TE and described their challenges with fitting into work and social environments when they felt they did not have the option to cover their head. The second participant who regretted having TE mentioned “the risks did not outweigh the rewards with my body's tendency to scar.”

## Discussion

This study identified a range of QOL outcomes, with the majority of patients showing an improved QOL following TE. Higher QOL scores were found to be associated with the scar and alopecia cohorts, as well as with an older age at start of treatment (*r* = 0.50, *P* = .029), shorter time in active treatment (*r* = −0.65, *P* < .005), fewer overall number of sessions (*r* = −0.54, *P* = .018), and a lower number of complications (*r* = −0.56, *P* = .013). The effect of TE on post-operative QOL is most likely related to multiple factors, with a trend towards improved QOL following less technically complex treatment courses. For example, a more complex lesion present at birth may require an earlier age of treatment, more time in treatment, and have more complications when compared to a less complex and acquired lesion.

Reconstruction of GCMN to address potential psychosocial concerns is one of the most common indications for TE. In our study, patients with GCMN had the lowest median QOL scores. Within this subgroup, lower QOL scores were associated with a greater number of sessions, younger age at start of treatment, longer time in active treatment, and an increased number of complications. Kinsler et al^12^ found that 11% to 14% of patients with large or giant CMNs felt their appearance had been worsened by surgical treatment, suggesting that there are patient- and treatment-related factors that impact post-operative QOL. These factors should be weighed in treatment decision making given that surgery is being carried out to improve the psychosocial wellbeing of patients rather than to reduce the risk of malignancy.^12–14^ For these reasons, Kinsler et al^15^ advocates for the use of a multidisciplinary team (pediatric dermatology, neuroradiology, and plastic surgery) in the management of these lesions, and for delaying of all routine surgeries for CMNs for at least the first year of life.

Patients with CMN on the head and neck were more likely to report that their surgery was worthwhile when compared to patients with lesions in other anatomic locations. This result supports the guidelines suggested by Kinsler and Bulstrode^15^ whereby patients with facial CMNs are offered reconstruction while observation is recommended for patients with lesions at other sites.

High complication rates have been described as inherent to the process of implantation and expansion of a foreign body. The present study found 68% of patients had one or more complications, with a negative correlation between number of complications and QOL following surgery. A wide range of complication rates for pediatric TE (7.5%-80%) have been described in the literature.^16–21^ Complication rates have been found to be higher for patients with TE in the extremities. Pandya et al found that 43% of patients aged 8 months to 58 years who underwent TE in the extremities experienced complications, whereas 27% of patients with TE in the non-extremities experienced complications.^22^ The lower extremity complication rate was 47%, while that of the upper extremity was 30%.^22^ A recent review of TE-related complication rates at our centre across a 10-year period found a 20.4% TE complication rate and a 25% flap complication rate following reconstruction.^6^ Given that a higher number of post-operative complications is associated with worse QOL, strategies to minimize complications, such as best practice guidelines and specialized multi-disciplinary clinics with individualized support for patients and families, should be established.^23^

The median total post-TE QOL score of +17 reported here is lower than that of similar studies examining QOL after a variety of different procedures, including mandibular distraction osteogenesis (+23.2) or tongue-lip adhesion (+19.4) in Pierre Robin sequence,^24^ otoplasty (+23.9-+24.2),^25–27^ and bone-anchored hearing aid fitting (+24.7).^28^ This may reflect the intensity of the TE process, commitment required, and high complication rates of such procedures.

Our qualitative results showed patient factors may contribute to QOL outcomes, such as individual perceptions of the scar resulting from treatment. Certain psychological factors, such as resilience, may play a role, but are difficult to predict or measure.

Although QOL outcomes in our study varied widely, 90% of participants would recommend TE to another child with a similar surgical indication, corroborating results from Logjes et al.^24^ This finding supports the value of TE despite its intensive and burdensome nature.

Our results are limited by the small and heterogeneous sample from a single centre, as well as participant recall bias. This survey-based study may also be limited by self-selection bias; we are unable to determine if patients who did not participate had different diagnostic characteristics, treatment details, and complications. Due to these limitations, the findings from this study should be interpreted with caution.

## Conclusion

Overall, we identified variable QOL after TE, with greater improvement in QOL associated with: treatment for scar and alopecia, an older age at treatment onset, less complex and shorter treatment courses, and fewer complications. Our findings and prior literature may suggest that children with GCMN undergoing TE in the extremities are at a greater risk for reduced QOL. Our preliminary descriptive findings may help guide providers during pre-operative planning and patient counselling and education. Health-related QOL following TE should be further explored with patient-reported outcome measures, with a matched cohort or prospective study design with pre- and post-treatment QOL measurements.

## Supplemental Material

sj-docx-1-psg-10.1177_22925503231217516 - Supplemental material for An Assessment of Quality-of-Life Following Tissue Expansion in Pediatric Patients Supplemental material, sj-docx-1-psg-10.1177_22925503231217516 for An Assessment of Quality-of-Life Following Tissue Expansion in Pediatric Patients by Dara Rykiss, Rebecca Courtemanche and Sally Hynes in Plastic Surgery

sj-jpg-2-psg-10.1177_22925503231217516 - Supplemental material for An Assessment of Quality-of-Life Following Tissue Expansion in Pediatric Patients Supplemental material, sj-jpg-2-psg-10.1177_22925503231217516 for An Assessment of Quality-of-Life Following Tissue Expansion in Pediatric Patients by Dara Rykiss, Rebecca Courtemanche and Sally Hynes in Plastic Surgery

sj-jpg-3-psg-10.1177_22925503231217516 - Supplemental material for An Assessment of Quality-of-Life Following Tissue Expansion in Pediatric Patients Supplemental material, sj-jpg-3-psg-10.1177_22925503231217516 for An Assessment of Quality-of-Life Following Tissue Expansion in Pediatric Patients by Dara Rykiss, Rebecca Courtemanche and Sally Hynes in Plastic Surgery
