# Supplementary material for: An Assessment of Quality-of-Life Following Tissue Expansion in Pediatric Patients
Source: Plast Surg (Oakv). 2023 Dec 12;33(2):289–96. doi: 10.1177/22925503231217516 (PMC12059438; doi:10.1177/22925503231217516)
Supplement: sj-docx-1-psg-10.1177_22925503231217516 - Supplemental material for An Assessment of Quality-of-Life Following Tissue Expansion in Pediatric Patients [file sj-docx-1-psg-10.1177_22925503231217516.docx]

**SUPPLEMENTAL FILE**

GLASGOW CHILDREN’S BENEFIT INVENTORY (GCBI)^10^

1. **Has your child’s operation made his/her/their overall life better or worse?**

Much worse (1) A little worse (2) No change (3) A little better (4) Much better (5)

1. **Has your child’s operation affected the things he/she/they does/do?**

Much worse (1) A little worse (2) No change (3) A little better (4) Much better (5)

1. **Has your child’s operation made his/her/their behaviour better or worse?**

Much worse (1) A little worse (2) No change (3) A little better (4) Much better (5)

1. **Has your child’s operation affected his/her/their progress and development?**

Much worse (1) A little worse (2) No change (3) A little better (4) Much better (5)

1. **Has your child’s operation affected how lively he/she/they is/are during the day?**

Much worse (1) A little worse (2) No change (3) A little better (4) Much better (5)

1. **Has your child’s operation affected how well he/she/they sleep(s) at night?**

Much worse (1) A little worse (2) No change (3) A little better (4) Much better (5)

1. **Has your child’s operation affected his/her/their enjoyment of food?**

Much less enjoyment (1) Less enjoyment (2) No change (3) More enjoyment (4) Much more enjoyment (5)

1. **Has your child’s operation affected how self-conscious he/she/they is/are with other people?**

Much less self-conscious (1) Less self-conscious (2) No change (3) More self-conscious (4) Much more self-conscious (5)

1. **Has your child’s operation affected how well he/she/they get(s) on with the rest of the family?**

Much worse (1) A little worse (2) No change (3) A little better (4) Much better (5)

1. **Has your child’s operation affected his/her/their ability to spend time and have fun with friends?**

Much worse (1) A little worse (2) No change (3) A little better (4) Much better (5)

1. **Has your child’s operation affected how embarrassed he/she/they is/are with other people?**

Much more embarrassed (1) More embarrassed (2) No change (3) Less embarrassed (4) Much less embarrassed (5)

1. **Has your child’s operation affected how easily distracted he/she/they has/have been?**

Much more easily distracted (1) More easily distracted (2) No change (3) Less easily distracted (4) Much less easily distracted (5)

1. **Has your child’s operation affected his/her/their learning?**

Much worse (1) A little worse (2) No change (3) A little better (4) Much better (5)

1. **Has your child’s operation affected the amount of time he/she/they has/have had to be off nursery, playgroup, or school?**

Much more time (1) More easily time (2) No change (3) Less time (4) Much less time (5)

1. **Has your child’s operation affected his/her/their ability to concentrate on a task?**

Much worse (1) A little worse (2) No change (3) A little better (4) Much better (5)

1. **Has your child’s operation affected how frustrated and irritable he/she/they is/are?**

Much more frustrated and irritable (1) More frustrated and irritable (2) No change (3) Less frustrated and irritable (4) Much less frustrated and irritable (5)

1. **Has your child’s operation affected how he/she/they feel(s) about himself/herself/them self?**

Much worse (1) A little worse (2) No change (3) A little better (4) Much better (5)

1. **Has your child’s operation affected how happy and content he/she/they is/are?**

Much less happy and content (1) Less happy and content (2) No change (3) More happy and content (4) Much more happy and content (5)

1. **Has your child’s operation affected his/her/their confidence?**

Much less confident (1) Less confident (2) No change (3) More confident (4) Much more confident (5)

1. **Has your child’s operation affected his/her/their ability to care for himself/herself/them self as well as you think they should, such as washing, dressing, and using the toilet?**

Much worse (1) A little worse (2) No change (3) A little better (4) Much better (5)

1. **Has your child’s operation affected his/her/their ability to enjoy leisure activities, such as swimming and sports, and general play?**

Much worse (1) A little worse (2) No change (3) A little better (4) Much better (5)

1. **Has your child’s operation affected how prone he/she/they is/are to catch colds or infections?**

Much more prone (1) More prone (2) No change (3) Less prone (4) Much less prone (5)

1. **Has your child’s operation affected how often he/she/they need(s) to visit a doctor?**

Much more often (1) More often (2) No change (3) Less often (4) Much less often (5)

1. **Has your child’s operation affected how much medication he/she/they has/have needed to take?**

Much more medication (1) More medication (2) No change (3) Less medication (4) Much less medication (5)

*Additional Question:* **Would you recommend tissue expansion to other children with the same diagnosis?**

Yes, No. Please explain, why of why not? __________________________________________________

**GLASGOW BENEFIT INVENTORY (GBI)^11^**

1. **Have the results of your operation affected the things you can do?**

Much worse (1) A little worse (2) No change (3) A little better (4) Much better (5)

1. **Have the results of your operation made your overall life better or worse?**

Much worse (1) A little worse (2) No change (3) A little better (4) Much better (5)

1. **Since your operation have you felt more or less optimistic about the future?**

Much less optimistic (1) Less optimistic (2) No change (3) More optimistic (4) Much more optimistic (5)

1. **Since your operation do you have more or less self-confidence?**

Much less self-confidence (1) Less self-confidence (2) No change (3) More self-confidence (4) Much more self-confidence (5)

1. **Since your operation do you feel better or worse about yourself?**

Much worse (1) A little worse (2) No change (3) A little better (4) Much better (5)

1. **Since your operation have you found it easier or harder to deal with company?**

Much harder (1) Harder (2) No change (3) Easier (4) Much easier (5)

1. **Since your operation do you feel more or less confident about job opportunities?**

Much less confident (1) Less confident (2) No change (3) More confident (4) Much more confident (5)

1. **Since your operation do you feel more or less embarrassed when with a group of people?**

Much more embarrassed (1) More embarrassed (2) No change (3) Less embarrassed (4) Much less embarrassed (5)

1. **Since your operation do you feel more or less self-conscious?**

Much less self-conscious (1) Less self-conscious (2) No change (3) More self-conscious (4) Much more self-conscious (5)

1. **Since your operation are you more or less inconvenienced by your (specific) problem?**

Much more inconvenienced (1) More inconvenienced (2) No change (3) Less inconvenienced (4) Much less inconvenienced (5)

1. **Since your operation have you been able to participate in more or fewer social situations?**

Many fewer activities (1) Fewer activities (2) No change (3) More activities (4) Many more activities (5)

1. **Since your operation have you been more or less inclined to withdraw from social situations?**

Much more inclined (1) More inclined (2) No change (3) Less inclined (4) Much less inclined (5)

1. **Since your operation do you feel you have more or less support from your friends?**

Much less support (1) Less support (2) No change (3) More support (4) Much more support (5)

1. **Since your operation do you feel you have more or less support from your family?**

Much less support (1) Less support (2) No change (3) More support (4) Much more support (5)

1. **Since your operation are there more or fewer people who really care about you?**

Many fewer people (1) Fewer people (2) No change (3) More people (4) Many more people (5)

1. **Since your operation have you been to your doctor, for any reason, more or less often?**

Much more often (1) More often (2) No change (3) Less often (4) Much less often (5)

1. **Since your operation have you had to take more or less medicine, for any reason?**

Much more medicine (1) More medicine (2) No change (3) Less medicine (4) Much less medicine (5)

1. **Since your operation, do you catch colds or infections more or less often?**

Much more often (1) More often (2) No change (3) Less often (4) Much less often (5)

*Additional Question:* **Would you recommend tissue expansion to other children with the same diagnosis?**

Yes, No. Please explain, why of why not? __________________________________________________
