# Supplementary figures and images for: An Assessment of Quality-of-Life Following Tissue Expansion in Pediatric Patients
Source: Plast Surg (Oakv). 2023 Dec 12;33(2):289–96. doi: 10.1177/22925503231217516 (PMC12059438; doi:10.1177/22925503231217516)

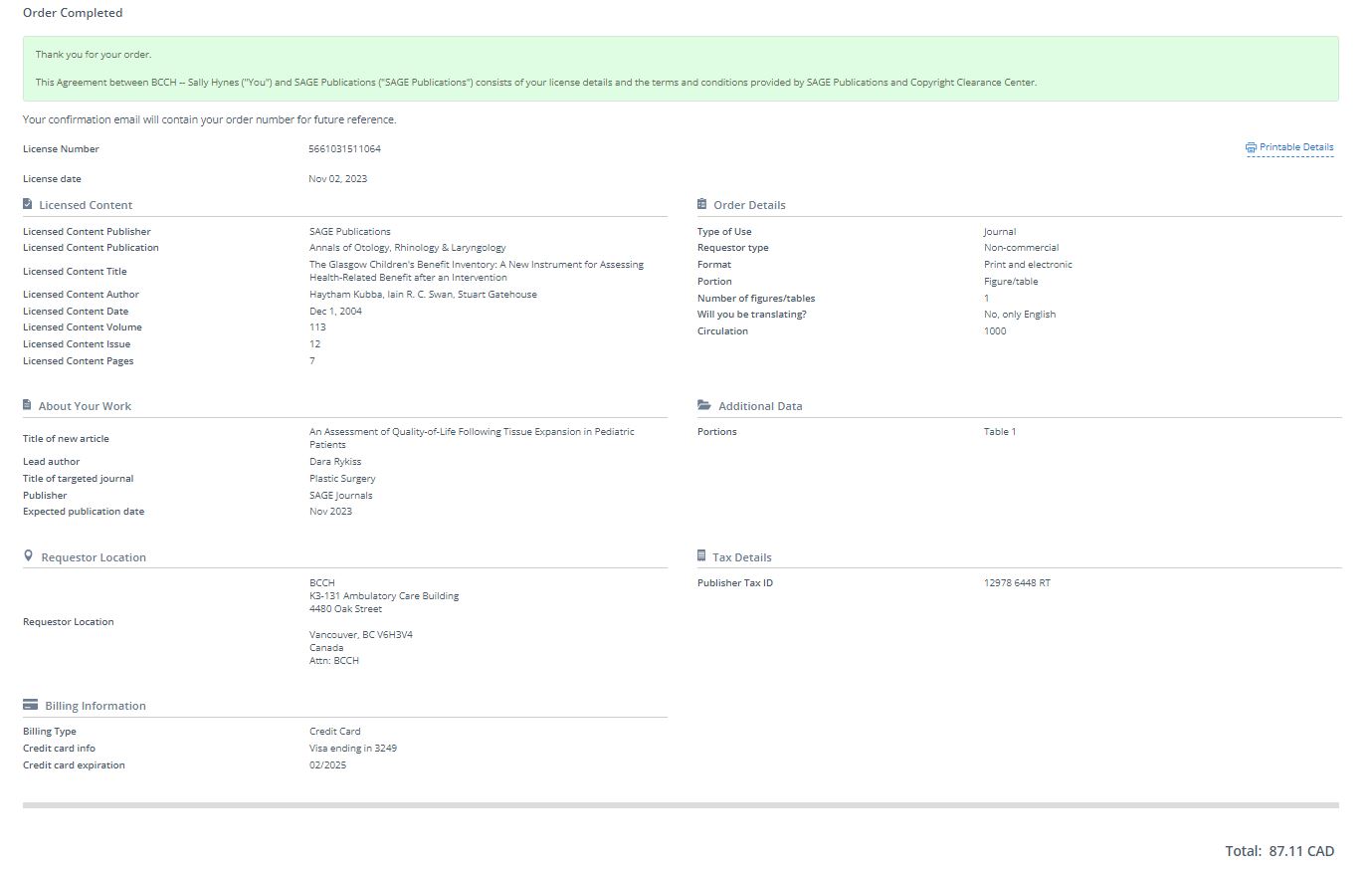

Supplement: sj-jpg-2-psg-10.1177_22925503231217516 - Supplemental material for An Assessment of Quality-of-Life Following Tissue Expansion in Pediatric Patients [file sj-jpg-2-psg-10.1177_22925503231217516.jpg]

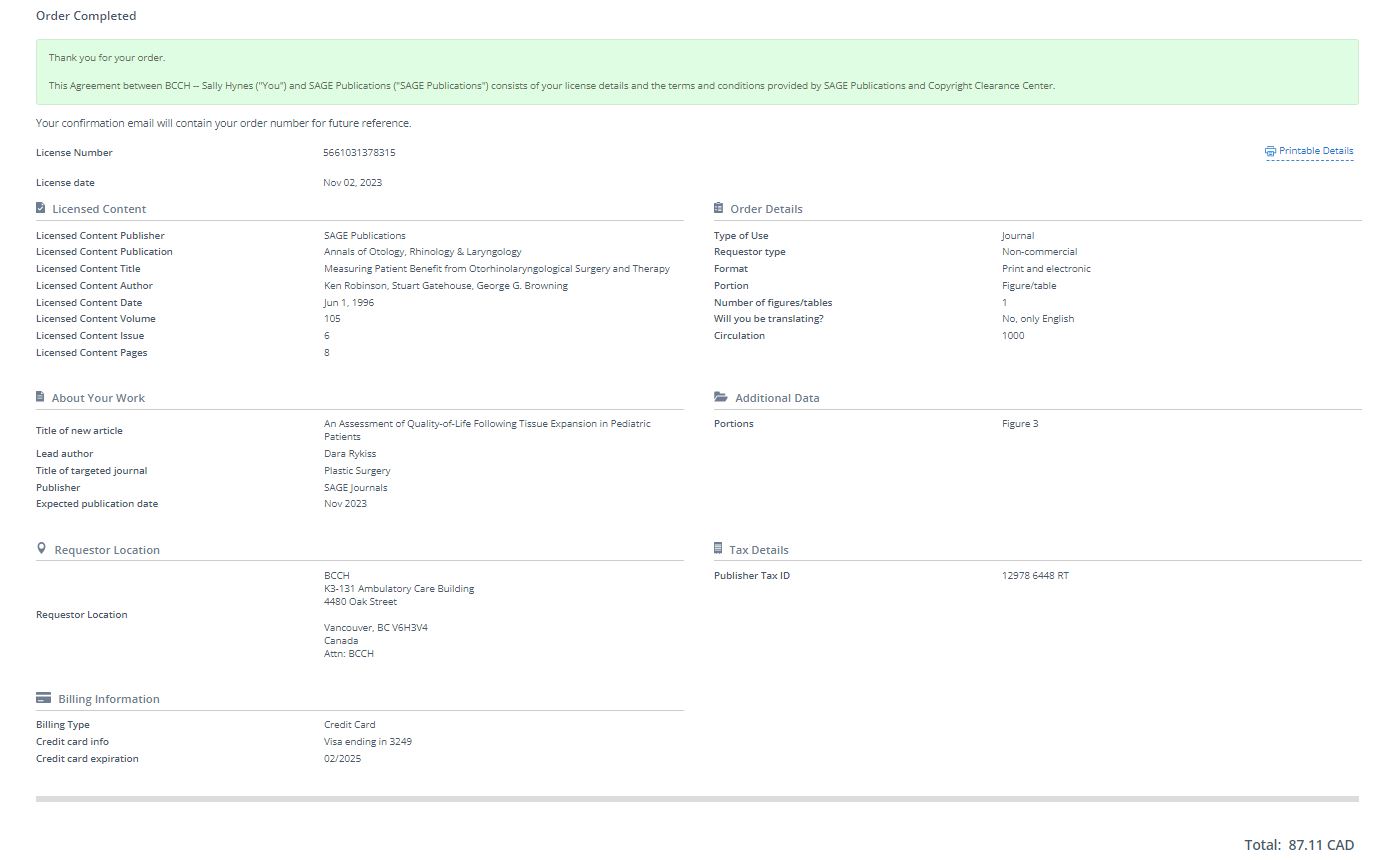

Supplement: sj-jpg-3-psg-10.1177_22925503231217516 - Supplemental material for An Assessment of Quality-of-Life Following Tissue Expansion in Pediatric Patients [file sj-jpg-3-psg-10.1177_22925503231217516.jpg]
